# Processed Food Contributions to Energy and Nutrient Intake Differ among US Children by Race/Ethnicity

**DOI:** 10.3390/nu7125503

**Published:** 2015-12-02

**Authors:** Heather A. Eicher-Miller, Victor L. Fulgoni, Debra R. Keast

**Affiliations:** 1 Department of Nutrition Science, Purdue University, 700 W. State Street, West Lafayette, IN 47907, USA; 2 Nutrition Impact, LLC, 9725 D Drive North, Battle Creek, MI 49014, USA; vic3rd@aol.com; 3 Food and Nutrition Database Research, Inc., 1801 Shadywood Lane, Okemos, MI 48864, USA; keastdeb@comcast.net

**Keywords:** processed food, dietary intake, nutrient intake, children, race, ethnicity

## Abstract

This study determined and compared the mean daily intake of energy and nutrients from processed foods by level of processing (minimally processed; processed for preservation, nutrient enhancement, and freshness; mixtures of combined ingredients; ready-to-eat processed foods; and prepared foods/meals) among non-Hispanic white, non-Hispanic black, and Mexican American US children. Data from participants 2–18 years old (*n* = 10,298) of the nationally representative cross-sectional National Health and Nutrition Examination Survey 2003–2008 with a complete one day, 24-h dietary recall were used to determine mean intake of energy and nutrients recommended for increase and decrease, as per the 2010 Dietary Guidelines for Americans, among child race/ethnic groups by category of food processing. Regression analysis was used to estimate and compare covariate-adjusted (gender, age, and poverty-income-level) least square means (*p* < 0.05/3 race/ethnic groups). All children, regardless of race or ethnicity consumed processed foods. Approximately 66% to 84% of total daily energy, saturated fat, cholesterol, fiber, total sugar, added sugars, calcium, vitamin D, potassium, and sodium intake are contributed by one of the five categories of processed foods. Clinicians and policy should primarily advise consideration of the energy and nutrient composition of foods, rather than the processing level, when selecting a healthy diet for children.

## 1. Introduction

Many US children fail to meet the current energy and nutrient recommendations outlined in the 2010 Dietary Guidelines for Americans (DGA) for the promotion of health, reduction of chronic disease risk, and prevalence of overweight and obesity [1,2]. Specifically, intakes of dietary fiber, calcium, vitamin D, and potassium tend to be suboptimal and should be increased while intakes of saturated fatty acids, added sugars, cholesterol and sodium tend to be excessive and should be decreased [1,2]. The 2010 DGA recommendations for children are based on the compilation of evidence establishing calcium and vitamin D intake to support optimal bone growth; fiber to promote healthy lipid profile, glucose tolerance, and normal gastrointestinal function; sodium and potassium to promote healthy blood pressure; saturated fatty acid and cholesterol intake to protect cardiovascular health; and minimal added sugar intake to balance energy consumption with expenditure and promote the intake of other essential nutrients [1,2].

The 2010 DGA recommendations for energy and nutrients are translated to the public by providing guidance on the intake of foods from certain food groups. However, the membership of a food to a specific food group is not based on nutrient composition or specific criteria, but rather, unspecified features of the food such as culture, origin, and culinary tradition. A food included in the “vegetable group” for example, lacks specific criteria to differentiate it from a food included in the “fruit group” or “grains group”. Processing (the deliberate changes made to a food between origin and consumption [3]) represents another feature of food. Recommendations for a healthy diet based on the level of food processing do not exist, but criteria to classify foods based on the physical, chemical, and sensory changes caused by processing have recently been completed by the International Food Information Council (IFIC) Foundation [4] in order to clarify the term “processing”, what foods are processed, and the purpose of processing. The foods within an IFIC Foundation category undergo a similar extent of change compared with their original unprocessed state. Foods categorized as “minimally processed” are minimally altered (e.g., packaged greens) and retain most of their inherent properties. “Foods processed for preservation, nutrient enhancement, and freshness” include canned and frozen foods while “mixtures of combined ingredients” include foods containing preservatives, flavors, colors, spices, sweeteners and oils to promote taste, safety, and visual appeal. “Ready-to-eat processed foods” include carbonated beverages, luncheon meats, breakfast cereals, yogurt, crackers, fruit drinks, and others. Lastly, the “prepared foods/meals” category includes foods packaged to preserve freshness and to ease preparation, such as prepared deli foods and frozen dinners [4]. Changes made to foods as a result of the extent of processing may be linked with nutrient contribution such that level of processing predicts more or less desirable nutrient intakes.

The energy and nutrient contributions of foods by food group to dietary intake are known to differ among race/ethnic groups of children [5,6,7,8,9,10,11] and suggest further intake differences may exist by processing level due to the changes foods undergo during processing. Foods categorized by level of processing have been shown to contribute differentially to adult and US energy and nutrient intake [12,13,14]; these categories may be useful parameters to consider when formulating dietary recommendations but intake differences among children currently remain unknown. This study was part of a greater effort to determine the contributions of processed foods to the dietary intake among diverse US populations [12,13,14], and is unique in its exclusive focus on children. Analysis was completed to determine the contributions of IFIC Foundation categories of processed foods [4] to the mean daily intake of energy, nutrients to increase, and nutrients to decrease, as specified by the 2010 DGA, and to compare the mean daily nutrient intakes of processed foods consumed by non-Hispanic white, non-Hispanic black, and Mexican American US children 2–18 years old. The hypothesis that specific processed food categories are responsible for differential energy and nutrient contributions among pediatric race/ethnic groups was tested.

## 2. Experimental Section 

### 2.1. Study Population and Study Design 

The National Health and Nutrition Examination Survey (NHANES) 2003–2004, 2005–2006, 2007–2008 are continuously conducted and nationally representative cross-sectional, population-based surveys of the National Center for Health Statistics of the Centers for Disease Control and Prevention [15]. The complex, multistage, probability sample represents the non-institutionalized, civilian, US population. Certain subpopulations were oversampled to allow reliable estimates of measures to be made among these groups. Participants were categorized according to age, sex, race-ethnicity and other characteristics as reported to a trained interviewer on a home-based questionnaire [16]. Classification of non-Hispanic white, non-Hispanic black, Mexican American, other Hispanic, and other race-ethnicity were made based on participant responses to questions regarding Hispanic/Latino identification and specific ancestral representation from a list including Mexican American and other Hispanic groups. Race was similarly self-identified from a list of 16 groups including “White” and “Black/African American” [16]. A health examination and 24-h dietary recall using the United States Department of Agriculture Automated, Multiple-Pass Method were completed at the NHANES Mobil Examination Center. A parent or guardian gave consent for children <18 years old while children ≥12 years old provided additional consent for certain portions of the interview. A parent or guardian acted as proxy for children 1 to 5 years old and children 6 to 11 years old were assisted by an adult [16]. All NHANES protocol and content was reviewed and approved [17]. Participants included 10,298 children 2–18 years old with a complete, reliable, 24-h dietary recall. 

### 2.2. Nutrients from Foods

Various United States Department of Agriculture food composition databases were used to derive nutrient and energy content of foods reported in the day one, 24-h dietary recall and have been previously described [12]. Energy (kcal) and nutrients of primary interest included: saturated fatty acids (gm), cholesterol (mg), total and added sugars (gm), dietary fiber (gm), calcium (mg), vitamin D (mcg), potassium (mg) and sodium (mg). 

### 2.3. Categorizing Processed Foods

Each reported food not from a restaurant or other eating establishment was evaluated separately and assigned to one of the following IFIC Foundation processed food categories: “minimally processed foods” (e.g., cow’s milk, washed and packaged fruits and vegetables, bagged salads, roasted nuts, coffee beans, raw meats, and eggs), “foods processed for preservation” (e.g., canned juice, canned and frozen vegetables and fruits, and soups made from canned vegetables or broth), “mixtures of combined ingredients” (e.g., breads, sweeteners, cheeses, dressings, condiments, margarine, creamers, cake and spice mixes), “ready-to-eat processed foods” (e.g., soft drinks, cereals, cookies, cakes, candy, juice drinks, yogurt, nut butters, frozen desserts, luncheon meats, beer, wine, and potato chips), or “prepared foods/meals” (e.g., pizza, chicken nuggets, frozen prepared meals, fish sticks, prepared deli foods, and corn dogs), based on the description provided in the food composition database. Foods originating from restaurants, schools, day care centers, soup kitchens, shelters or other eating establishments were grouped together in analysis and designated “foods from restaurants/cafeterias” (representing 22.2%, 26.1%, and 22.8% of the number of foods for non-Hispanic white, non-Hispanic black, and Mexican American children, respectively, see Table 1, Table 2 and Table 3) because inadequate information was available to assign them to one of the IFIC Foundation categories. Foods often made at home from other processed ingredients such as pasta salad (*i.e.*, pasta and dressing are both “mixtures of combined ingredients”) were classified as “ready-to-eat processed foods”. Ready-to-serve, canned, and condensed soups were assigned to “mixtures of combined ingredients”, while homemade soups were classified as “minimally processed foods”. Soups made from pre-made broth or other canned foods were classified to a higher level of processing: “foods processed for preservation” (see Supplemental Table S1 in reference [13] for a list of foods by processed food category [13]).

**Table 1:** Mean (SEM) intake and percent contribution (SEM) of International Food Information Council Foundation processed food categories to the number of foods and total daily intake of energy and selected nutrients by non-Hispanic white children 2–18 years participating in NHANES 2003–2008 (*n* = 2954) ^a^.

Nutrients		Number of Foods	Energy	Saturated Fatty Acids	Cholesterol	Na	Total Sugar	Added Sugars	Ca	Vitamin D	K	Fiber
Units of Nutrient Intake			Kcal	g	Mg	mg	g	g	mg	µg	mg	g
All foods	Mean intake	15.1 (0.2)	2064 (23)	27.4 (0.4)	221.4 (4.8)	3176 (55)	142.5 (1.9)	92.7 (1.9)	1059 (17)	5.9 (0.2)	12.8 (0.2)	12.8 (0.2)
	% contribution ^b,c^	100	100	100	100	100	100	100	100	100	100	100
IFIC category foods ^d^	Mean intake	11.7 (0.2)	1457 (22)	18.6 (0.4)	152.3 (3.8)	2133 (45)	111.0 (2.0)	69.3 (1.7)	795 (19)	4.9 (0.2)	9.3 (0.2)	9.3 (0.2)
	% contribution ^b,c^	77.8 (0.8)	70.6 (0.9)	67.8 (1.1)	68.8 (1.2)	67.2 (1.1)	77.9 (0.8)	74.8 (0.9)	75.1 (1.2)	84.1 (1.4)	72.2 (1.0)	72.2 (1.0)
1. Minimally processed foods	Mean intake	3.4 (0.1)	252 (9)	4.3 (0.2)	64.6 (3.0)	250 (10)	20.4 (0.7)	1.3 (0.3)	333 (12)	3.2 (0.1)	1.9 (0.1)	1.9 (0.1)
	% contribution ^b,c^	22.8 (0.5)	12.2 (0.4)	15.5 (0.6)	29.2 (1.2)	7.9 (0.3)	14.3 (0.5)	1.4 (0.3)	31.4 (0.9)	54.1 (1.4)	14.8 (0.6)	14.8 (0.6)
2. Foods processed for preservation	Mean intake	0.6 (0.0)	59 (2)	0.1 (0.0)	0.4 (0.1)	36 (3)	11.1 (0.5)	0.6 (0.1)	34 (3)	0.1 (0.0)	0.6 (0.0)	0.6 (0.0)
	% contribution ^b,c^	3.9 (0.1)	2.8 (0.1)	0.3 (0.1)	0.2 (0.0)	1.1 (0.1)	7.8 (0.4)	0.6 (0.1)	3.2 (0.2)	1.0 (0.2)	5.0 (0.3)	5.0 (0.3)
3. Mixtures of combined ingredients	Mean intake	2.5 (0.1)	298 (9)	3.9 (0.2)	27.5 (1.5)	604 (18)	11.8 (0.5)	8.9 (0.5)	154 (5)	0.3 (0.0)	2.0 (0.1)	2.0 (0.1)
	% contribution ^b,c^	16.4 (0.3)	14.4 (0.4)	14.2 (0.5)	12.4 (0.6)	19.0 (0.6)	8.3 (0.4)	9.6 (0.5)	14.5 (0.5)	5.0 (0.3)	15.9 (0.7)	15.9 (0.7)
4. Ready-to-eat processed foods	Mean intake	5.0 (0.1)	751 (16)	8.8 (0.3)	45.8 (1.7)	1007 (25)	66.6 (2.0)	58.1 (1.8)	236 (9)	1.3 (0.1)	4.1 (0.1)	4.1 (0.1)
	% contribution ^b,c^	32.9 (0.6)	36.4 (0.8)	32.1 (0.9)	20.7 (0.8)	31.7 (0.8)	46.7 (1.1)	62.7 (1.2)	22.2 (0.7)	22.7 (1.0)	31.9 (0.9)	31.9 (0.9)
5. Prepared foods/meals	Mean intake	0.3 (0.0)	98 (7)	1.6 (0.1)	14.0 (1.2)	236 (19)	1.1 (0.1)	0.5 (0.1)	39 (4)	0.1 (0.0)	0.6 (0.1)	0.6 (0.1)
	% contribution ^b,c^	1.7 (0.1)	4.7 (0.3)	5.7 (0.4)	6.3 (0.5)	7.4 (0.5)	0.8 (0.1)	0.5 (0.1)	3.7 (0.4)	1.3 (0.2)	4.6 (0.4)	4.6 (0.4)
Foods from restaurants/Cafeterias	Mean intake	3.4 (0.1)	608 (22)	8.8 (0.3)	69.2 (3.4)	1042 (44)	31.5 (1.2)	23.4 (0.9)	263 (13)	0.9 (0.1)	3.6 (0.1)	3.6 (0.1)
	% contribution ^b,c^	22.2 (0.8)	29.4 (0.9)	32.2 (1.1)	31.2 (1.2)	32.8 (1.1)	22.1 (0.8)	25.2 (0.9)	24.9 (1.2)	15.9 (1.4)	27.8 (1.0)	27.8 (1.0)

Note: IFIC, International Food Information Council. SEM, Standard error of the mean. ^a^ Application of proper sample weights and adjustment for the complex sample design allow inference to the non-institutionalized US population; ^b^ Total numbers do not always add to sample size due to missing values; percents do not always add to 100 due to rounding; ^c^ Percent contribution of specified food category to the total daily intake of that particular dietary component; ^d^ IFIC Category foods include all foods not from restaurants/cafeterias.

**Table 2:** Mean (SEM) intake and percent contribution (SEM) of International Food Information Council Foundation processed food categories to the number of foods and total daily intake of energy and selected nutrients by non-Hispanic black children 2–18 years participating in NHANES 2003–2008 (*n* = 3139) ^a^.

Nutrients		Number of Foods	Energy	Saturated Fatty Acids	Cholesterol	Na	Total Sugar	Added Sugars	Ca	Vitamin D	K	Fiber
Units of Nutrient Intake			Kcal	g	Mg	mg	g	g	mg	µg	mg	g
All foods	Mean intake	13.8 (0.1)	1961 (26)	25.1 (0.4)	226.3 (5.7)	3064 (46)	131.5 (2.4)	85.4 (2.0)	829 (13)	4.4 (0.1)	2025 (29)	11.4 (0.2)
	% contribution ^b,c^	100	100	100	100	100	100	100	100	100	100	100
IFIC category foods ^d^	Mean intake	10.2 (0.2)	1363 (34)	16.5 (0.5)	153.9 (5.5)	2045 (57)	101.7 (3.1)	66.9 (2.3)	565 (17)	3.2 (0.1)	1399 (40)	7.9 (0.2)
	% contribution ^b,c^	73.9 (1.5)	69.5 (1.5)	65.7 (1.6)	68.0 (1.7)	66.7 (1.5)	77.3 (1.6)	78.3 (1.5)	68.2 (1.6)	72.0 (2.3)	69.1 (1.7)	69.7 (1.6)
1. Minimally processed foods	Mean intake	2.6 (0.1)	210 (7)	3.8 (0.1)	70.0 (3.7)	232 (10)	11.5 (0.4)	0.4 (0.1)	185 (6)	1.8 (0.1)	418 (14)	1.4 (0.1)
	% contribution ^b,c^	18.9 (0.5)	10.7 (0.4)	15.0 (0.5)	30.9 (1.2)	7.6 (0.3)	8.8 (0.3)	0.5 (0.1)	22.3 (0.7)	40.2 (1.5)	20.6 (0.7)	12.0 (0.5)
2. Foods processed for preservation	Mean intake	0.7 (0.0)	69 (4)	0.1 (0.0)	0.4 (0.1)	42 (4)	13.0 (0.8)	0.7 (0.1)	48 (4)	0.1 (0.0)	199 (11)	0.7 (0.0)
	% contribution ^b,c^	4.8 (0.2)	3.5 (0.2)	0.4 (0.1)	0.2 (0.0)	1.4 (0.1)	9.9 (0.5)	0.8 (0.1)	5.8 (0.5)	1.2 (0.2)	9.8 (0.5)	6.3 (0.4)
3. Mixtures of combined ingredients	Mean intake	2.1 (0.1)	287 (11)	3.4 (0.2)	28.1 (2.5)	611 (24)	11.3 (0.5)	8.4 (0.5)	131 (7)	0.3 (0.0)	194 (10)	2.0 (0.1)
	% contribution ^b,c^	15.6 (0.5)	14.6 (0.5)	13.5 (0.6)	12.4 (1.0)	19.9 (0.7)	8.6 (0.4)	9.8 (0.6)	15.9 (0.8)	6.2 (0.5)	9.6 (0.5)	17.3 (0.8)
4. Ready-to-eat processed foods	Mean intake	4.5 (0.1)	711 (19)	7.8 (0.3)	43.2 (2.1)	958 (30)	64.9 (2.3)	57.0 (2.1)	171 (7)	1.0 (0.0)	515 (18)	3.4 (0.1)
	% contribution ^b,c^	32.9 (0.8)	36.2 (0.9)	31.1 (1.0)	19.1 (1.0)	31.3 (0.9)	49.3 (1.2)	66.8 (1.4)	20.7 (0.7)	22.7 (1.0)	25.4 (0.8)	30.0 (0.9)
5. Prepared foods/meals	Mean intake	0.2 (0.0)	86 (7)	1.4 (0.1)	12.3 (1.1)	202 (17)	1.0 (0.1)	0.4 (0.0)	30 (3)	0.1 (0.0)	73 (6)	0.5 (0.0)
	% contribution ^b,c^	1.8 (0.1)	4.4 (0.3)	5.7 (0.5)	5.4 (0.5)	6.6 (0.5)	0.7 (0.1)	0.5 (0.0)	3.6 (0.4)	1.7 (0.3)	3.6 (0.3)	4.2 (0.3)
Foods from restaurants/Cafeterias	Mean intake	3.6 (0.2)	598 (30)	8.6 (0.4)	72.4 (4.3)	1020 (49)	29.9 (2.0)	18.5 (1.3)	264 (14)	1.2 (0.1)	625 (35)	3.5 (0.2)
	% contribution ^b,c^	26.1 (1.5)	30.5 (1.5)	34.3 (1.6)	32.0 (1.7)	33.3 (1.5)	22.7 (1.6)	21.7 (1.5)	31.8 (1.6)	28.0 (2.3)	30.9 (1.7)	30.3 (1.6)

Note: IFIC, International Food Information Council. SEM, Standard error of the mean. ^a^ Application of proper sample weights and adjustment for the complex sample design allow inference to the non-institutionalized US population; ^b^ Total numbers do not always add to sample size due to missing values; percents do not always add to 100 due to rounding; ^c^ Percent contribution of specified food category to the total daily intake of that particular dietary component; ^d^ IFIC Category foods include all foods not from restaurants/cafeterias.

**Table 3:** Mean (SEM) intake and percent contribution (SEM) of International Food Information Council Foundation processed food categories to the number of foods and total daily intake of energy and selected nutrients by Mexican-American children 2–18 years participating in NHANES 2003–2008. (*n* = 3061) ^a^.

Nutrients		Number of Foods	Energy	Saturated Fatty Acids	Cholesterol	Na	Total Sugar	Added Sugars	Ca	Vitamin D	K	Fiber
Units of Nutrient Intake			Kcal	g	mg	mg	g	g	mg	µg	mg	g
All foods	Mean intake	14.9 (0.1)	1953 (30)	24.9 (0.4)	247.1 (6.0)	2887 (42)	130.4 (2.7)	77.4 (2.7)	1001 (21)	5.8 (0.2)	2308 (44)	14.1 (0.3)
	% contribution ^b,c^	100	100	100	100	100	100	100	100	100	100	100
IFIC category foods ^d^	Mean intake	11.5 (0.2)	1403 (24)	17.0 (0.3)	183.3 (5.5)	1955 (33)	101.9 (2.3)	59.2 (2.0)	742 (20)	4.7 (0.2)	1736 (44)	10.7 (0.3)
	% contribution ^b,c^	77.2 (0.9)	71.8 (0.9)	68.5 (0.8)	74.2 (0.9)	67.7 (0.9)	78.1 (0.9)	76.4 (0.9)	74.1 (1.1)	80.7 (1.1)	75.2 (1.0)	75.6 (1.0)
1. Minimally processed foods	Mean intake	3.9 (0.1)	327 (13)	5.4 (0.2)	100.7 (5.1)	366 (15)	21.3 (0.9)	0.4 (0.1)	317 (13)	3.1 (0.1)	777 (30)	3.2 (0.2)
	% contribution ^b,c^	26.0 (0.7)	16.8 (0.6)	21.8 (0.9)	40.8 (1.6)	12.7 (0.6)	16.3 (0.7)	0.6 (0.1)	31.7 (1.0)	52.8 (1.2)	33.7 (0.9)	22.3 (0.8)
2. Foods processed for preservation	Mean intake	0.6 (0.0)	60 (4)	0.0 (0.0)	0.2 (0.1)	19 (3)	12.0 (0.9)	0.8 (0.3)	54 (5)	0.1 (0.0)	179 (11)	0.5 (0.1)
	% contribution ^b,c^	3.9 (0.2)	3.1 (0.2)	0.2 (0.0)	0.1 (0.0)	0.6 (0.1)	9.2 (0.7)	1.0 (0.4)	5.4 (0.5)	1.2 (0.2)	7.8 (0.4)	3.2 (0.4)
3. Mixtures of combined ingredients	Mean intake	2.6 (0.1)	354 (12)	4.1 (0.2)	41.8 (2.8)	685 (21)	12.8 (0.7)	8.8 (0.6)	173 (9)	0.5 (0.0)	286 (12)	3.2 (0.1)
	% contribution ^b,c^	17.7 (0.4)	18.1 (0.6)	16.7 (0.7)	16.9 (1.0)	23.7 (0.6)	9.8 (0.6)	11.3 (0.8)	17.3 (0.7)	7.9 (0.6)	12.4 (0.4)	22.7 (0.7)
4. Ready-to-eat processed foods	Mean intake	4.3 (0.1)	620 (14)	6.8 (0.2)	34.7 (1.4)	793 (18)	55.4 (1.9)	49.0 (1.8)	184 (6)	1.1 (0.0)	459 (12)	3.6 (0.1)
	% contribution ^b,c^	28.7 (0.5)	31.8 (0.6)	27.2 (0.9)	14.0 (0.7)	27.4 (0.6)	42.5 (1.0)	63.3 (0.9)	18.4 (0.7)	18.2 (0.7)	19.9 (0.6)	25.9 (0.7)
5. Prepared foods/meals	Mean intake	0.1 (0.0)	41 (4)	0.7 (0.1)	5.9 (0.6)	93 (10)	0.4 (0.1)	0.2 (0.0)	14 (2)	0.0 (0.0)	35 (3)	0.2 (0.0)
	% contribution ^b,c^	0.8 (0.1)	2.1 (0.2)	2.6 (0.3)	2.4 (0.3)	3.2 (0.4)	0.3 (0.0)	0.2 (0.0)	1.4 (0.2)	0.5 (0.1)	1.5 (0.2)	1.6 (0.2)
Foods from restaurants/Cafeterias	Mean intake	3.4 (0.1)	551 (20)	7.8 (0.3)	63.8 (2.4)	932 (31)	28.5 (1.5)	18.2 (1.1)	259 (11)	1.1 (0.1)	572 (24)	3.4 (0.1)
	% contribution ^b,c^	22.8 (0.9)	28.2 (0.9)	31.5 (0.8)	25.8 (0.9)	32.3 (0.9)	21.9 (0.9)	23.6 (0.9)	25.9 (1.1)	19.3 (1.1)	24.8 (1.0)	24.4 (1.0)

Note: IFIC, International Food Information Council. SEM, Standard error of the mean. ^a^ Application of proper sample weights and adjustment for the complex sample design allow inference to the non-institutionalized US population; ^b^ Total numbers do not always add to sample size due to missing values; percents do not always add to 100 due to rounding; ^c^ Percent contribution of specified food category to the total daily intake of that particular dietary component; ^d^ IFIC Category foods include all foods not from restaurants/cafeterias.

### 2.4. Estimation of Mean Intake and Comparison of Population Based Groups

Mean total energy and nutrient intake and mean intake from each IFIC Foundation category, were estimated for all participants based on the 24-h recall, after excluding those with incomplete dietary recalls. The respondent level totals were summed across the sample population to determine total energy and nutrients consumed from each IFIC Foundation category. Calculated statistics include means, percent contribution provided by each IFIC Foundation category (total nutrient *i* provided by category *j*/total nutrient *i* provided by all foods × 100 where *i* = energy and nutrients detailed above and *j* = IFIC Foundation categories) and standard errors. Comparisons of mean daily energy/nutrient intake, and energy/nutrient intakes from each IFIC Foundation category by gender, income, and race/ethnic groups were completed using regression analysis to generate covariate adjusted least square means and were considered statistically significant when *p* < 0.05/3 (race/ethnic groups, Bonferronni type adjustment for multiple comparisons of sub-groups) among non-Hispanic whites (*n* = 2954), non-Hispanic blacks (*n* = 3139), and Mexican Americans (*n* = 3061). “Other Hispanic” and “other race/ethnic” groups were not sampled in a way to be nationally representative and thus, excluded. Energy intake and age were included as continuous variables. Gender (male, female) and poverty-income-ratio (PIR) category, calculated as household income divided by the federal poverty guideline for household income, (PIR 1.85 or less, PIR > 1.85, PIR missing) were included categorically. Analyses were completed using PROC REGRESS, PROC RATIO and other related procedures of SUDAAN Release 10.0.1 (Research Triangle Institute, Research Triangle Park, NC, USA) with proper sample weighting and adjustment for the complex design.

## 3. Results 

### 3.1. Contributions of Processed Food Categories to Energy and Selected Nutrients

All processed food categories contributed nutrients to increase (fiber, calcium, vitamin D, and potassium) or decrease (saturated fatty acids, cholesterol, total and added sugars, and sodium) in the daily dietary intake of US children 2–18 years old (Table 1, Table 2 and Table 3, Supplemental Figure S1). 

“Foods processed for preservation” and “prepared foods/meals” made minimal contributions to energy and most nutrient intakes (except sometimes Na and total sugars) compared with the “ready-to-eat processed foods,” “mixtures of combined ingredients” and “minimally processed foods” categories. Thus, results will focus on the energy and nutrient contributions made by these categories. “Minimally processed foods” made proportionally high contributions (compared with energy 11%–17%) to mean cholesterol (29%–41%), calcium (22%–32%), vitamin D (40%–54%), potassium (15%–34%), and fiber (12%–22%), while making low contributions to added sugar (1%). The “mixtures of combined ingredients” category contributions to sodium (19%–24%) and fiber (16%–23%) were high in proportion to energy (14%–18%), while the contribution to total sugars (8%–10%), vitamin D (5%–8%) and potassium (10%–16%) were low. Contributions of the “ready-to-eat processed foods” were also diverse: mean cholesterol (14%–21%), calcium (18%–22%), vitamin D (18%–23%), and potassium (20%–32%) were lower than mean energy contributions (32%–36%), while total (43%–49%) and added sugars (63%–67%) were higher.

### 3.2. Comparisons of Race/Ethnic Groups’ Adjusted Mean Energy and Nutrient Intake

Differences among race/ethnic groups were numerous (Table 4, Supplemental Figure S2); as such, the results and discussion below will focus on significant energy and nutrient intake differences among race/ethnic groups when “all foods” and “IFIC Foundation category” intake differences existed along with specific processed food category differences when controlling for covariates.

**Table 4:** Comparison of covariate-adjusted mean (SEM) intake of energy, selected nutrients, and percentage of population consuming food from International Food Information Council Foundation processed food categories by race/ethnicity in children 2–18 years as drawn from NHANES 2003–2008 ^a^.

Category	Race ^b^	Population Consuming Food, % ^c^	Energy	Saturated Fatty Acids	Cholesterol	Na	Total Sugar	Added Sugars	Ca	Vitamin D	K	Fiber
			Kcal ^c^	g ^d^	mg ^d^	mg ^d^	g ^d^	g ^d^	mg ^d^	µg ^d^	mg ^d^	g ^d^
All foods	White	100	2058 (18) ^x^	27.0 (0.2) ^x^	217.7 (3.6) ^x^	3116 (30) ^x,y^	140.5 (1.3) ^x^	90.9 (1.6) ^x^	1044 (12) ^x^	5.8 (0.1)^x^	2222 (30) ^x^	12.7 (0.2) ^x^
	Black	100	1960 (28) ^y^	26.0 (0.2) ^y^	234.1 (5.2) ^y^	3183 (29) ^x^	136.1 (1.6) ^x,y^	88.8 (1.6) ^x^	866 (12) ^y^	4.5 (0.1) ^y^	2076 (18) ^y^	11.8 (0.2) ^y^
	MA	100	1982 (31) ^x,y^	25.8 (0.2) ^y^	255.9 (6.3) ^z^	3032 (39) ^y^	134.8 (1.7) ^y^	82.0 (2.1) ^y^	1030 (19) ^x^	5.8 (0.2) ^x^	2348 (32) ^z^	14.5 (0.2) ^z^
IFIC category foods ^e^	White	99.6 (0.1)	1455 (21)	18.4 (0.4)	150.8 (3.7) ^x^	2102 (40)	109.6 (1.8)	68.2 (1.6) ^x^	783 (17) ^x^	4.9 (0.2) ^x^	1644 (34) ^x^	9.2 (0.2) ^x^
	Black	99.6 (0.1)	1363 (36)	17.0 (0.4)	157.8 (5.7) ^x^	2114 (52)	105.2 (2.8)	69.1 (2.1) ^x^	597 (15) ^y^	3.3 (0.1) ^y^	1438 (36) ^y^	8.2 (0.2) ^y^
	MA	99.7 (0.1)	1412 (24)	17.4 (0.2)	186.1 (5.8) ^y^	2023 (38)	104.8 (1.7)	61.9 (1.6) ^y^	763 (21) ^x^	4.8 (0.2) ^x^	1757 (40) ^x^	10.8 (0.3) ^z^
1. Minimally processed foods	White	90.6 (0.1) ^x^	255 (8) ^x^	4.3 (0.2) ^x^	64.7 (2.8) ^x^	252 (10) ^x^	20.3 (0.7) ^x^	1.4 (0.3) ^x^	329 (12) ^x^	3.2 (0.1) ^x^	659 (23) ^x^	1.9 (0.1^)x^
	Black	85.8 (1.1) ^y^	203 (8) ^y^	3.7 (0.1) ^y^	70.0 (4.1) ^x^	229 (11) ^x^	11.9 (0.5) ^y^	0.3 (0.1) ^y^	194 (6) ^y^	1.9 (0.1) ^y^	424 (15) ^y^	1.4 (0.1) ^y^
	MA	93.0 (0.8) ^x^	319 (12) ^z^	5.3 (0.2) ^z^	100.0 (5.4) ^y^	362 (15) ^y^	21.3 (0.9) ^x^	0.4 (0.2) ^y^	323 (14) ^x^	3.1 (0.1) ^x^	775 (29) ^z^	3.1 (0.1) ^z^
2. Foods processed for preservation	White	39.5 (1.2)	60 (2)	0.1 (0.0) ^x^	0.4 (0.1)	37 (3) ^x^	11.1 (0.5) ^x^	0.6 (0.1)	34 (3) ^x^	0.1 (0.0)	163 (6) ^x^	0.6 (0.0) ^x^
	Black	41.2 (1.3)	69 (4)	0.1 (0.0) ^x^	0.4 (0.1)	40 (4) ^x^	13.2 (0.7) ^y^	0.7 (0.1)	49 (4) ^y^	0.1 (0.0)	202 (10) ^y^	0.7 (0.0) ^x^
	MA	36.6 (1.5)	57 (4)	0.0 (0.0) ^y^	0.2 (0.1)	15 (3) ^y^	11.6 (0.8) ^x,y^	0.8 (0.3)	52 (5) ^y^	0.1 (0.0)	175 (11) ^x,y^	0.4 (0.1) ^y^
3. Mixtures of combined ingredients	White	80.1 (0.9) ^x^	296 (9) ^x^	3.8 (0.2) ^x,y^	26.9 (1.4) ^x^	593 (18) ^x^	11.6 (0.5)	8.7 (0.5)	151 (5) ^x^	0.3 (0.0) ^x^	192 (8) ^x^	2.0 (0.1) ^x^
	Black	76.6 (1.4) ^x^	288 (11) ^x^	3.5 (0.2) ^x^	29.4 (2.7) ^x^	633 (24) ^x^	11.7 (0.5)	8.7 (0.5)	138 (7) ^x^	0.3 (0.0) ^x^	201 (10) ^x^	2.0 (0.1) ^x^
	MA	86.3 (1.0) ^y^	359 (13) ^y^	4.3 (0.2) ^y^	42.9 (2.7) ^y^	711 (22) ^y^	13.2 (0.8)	9.1 (0.6)	178 (9)^y^	0.5 (0.0) ^y^	292 (12) ^y^	3.3 (0.1) ^y^
4. Ready-to-eat processed foods	White	98.3 (0.3)	746 (16) ^x^	8.6 (0.3) ^x^	44.9 (1.6) ^x^	988 (24) ^x^	65.4 (1.9) ^x^	57.1 (1.7) ^x,y^	231 (8) ^x^	1.3 (0.1) ^x^	549 (13) ^x^	4.0 (0.1) ^x^
	Black	97.2 (0.4)	716 (19) ^x^	8.2 (0.2) ^x^	45.3 (2.2) ^x^	1001 (27) ^x^	67.3 (2.1) ^x^	59.0 (2.0) ^x^	183 (7) ^y^	1.1 (0.0) ^y^	535 (16) ^x^	3.6 (0.1) ^y^
	MA	97.3 (0.3)	634 (16) ^y^	7.1 (0.2) ^y^	36.7 (1.5) ^y^	833 (21) ^y^	58.2 (1.7) ^y^	51.5 (1.6) ^y^	192 (6) ^y^	1.1 (0.0) ^y^	476 (11) ^y^	3.8 (0.1) ^x,y^
5. Prepared foods/meals	White	22.8 (1.3) ^x^	97 (8) ^x^	1.5 (0.1) ^x^	13.8 (1.1) ^x^	232 (18) ^x^	1.1 (0.1) ^x^	0.5 (0.0) ^x^	38 (4) ^x^	0.1 (0.0) ^x^	81 (6) ^x^	0.6 (0.1) ^x^
	Black	21.9 (1.3) ^x^	87 (7) ^x^	1.5 (0.1) ^x^	12.6 (1.1) ^x^	211 (17) ^x^	1.0 (0.1) ^x^	0.4 (0.0) ^x^	32 (3) ^x^	0.1 (0.0) ^x^	76 (6) ^x^	0.5 (0.0) ^x^
	MA	10.4 (0.9) ^y^	43 (5) ^y^	0.7 (0.1) ^y^	6.3 (0.6) ^y^	103 (13) ^y^	0.5 (0.1) ^y^	0.2 (0.0) ^y^	17 (3) ^y^	0.0 (0.0) ^y^	38 (4) ^y^	0.3 (0.0) ^y^
Foods from restaurants/cafeterias	White	65.2 (1.5)	604 (23)	8.6 (0.3)	66.9 (2.8)	1014 (40)	30.9 (1.1)	22.7 (0.8)	260 (13)	1.0 (0.1)	578 (26)	3.5 (0.1)
	Black	65.2 (2.2)	597 (30)	9.0 (0.4)	76.3 (4.1)	1069 (48)	30.9 (2.0)	19.7 (1.2)	270 (15)	1.2 (0.1)	638 (34)	3.6 (0.2)
	MA	65.9 (1.5)	570 (22)	8.4 (0.2)	69.8 (2.5)	1008 (29)	30.0 (1.4)	20.1 (1.0)	267 (10)	1.1 (0.1)	592 (22)	3.6 (0.1)

Note: IFIC, International Food Information Council. SEM, Standard error of the mean. ^a^ Significant differences (*p* < 0.05/3 race/ethnic groups, Bonferronni type adjustment for multiple comparisons of sub-groups) within each category of foods are indicated by differing lower-case alphabetic superscript (x,y,z); significant differences are not established for race/ethnic sub-groups with a similar lower-case alphabetic superscript. Survey weights and adjustment for the complex survey design were properly applied allowing inference to the non-institutionalized US population; ^b^ Non-Hispanic white (White) *n* = 2954, non-Hispanic black (Black) *n* = 3139, and Mexican-American (MA) *n* = 3061; ^c^ Covariates include gender, age (year), and poverty-income-ratio; ^d^ Covariates include energy (Kcal), age (year), gender, and poverty-income-ratio; ^e^ IFIC Foundation foods include all foods not from restaurants or cafeterias.

The results and discussion will also focus mainly on processed food categories that contributed at least 10% of energy in one of the three race/ethnic groups. Mexican American children consumed greater energy, cholesterol, calcium, vitamin D, potassium, and fiber from the “minimally processed foods” compared with non-Hispanic black and sometimes also non-Hispanic white children. In addition, a greater proportion of Mexican American and non-Hispanic white children consumed “minimally processed foods” compared with non-Hispanic black children. However, mean added sugar intake was lower for Mexican American and non-Hispanic black compared with non-Hispanic white children from “minimally processed foods”. A greater percentage of Mexican American children consumed foods from the “mixtures of combined ingredients” category while also consuming the most energy, cholesterol, calcium, vitamin D, potassium, and fiber from that category compared with non-Hispanic white and black children. The “ready-to-eat processed foods” category derived energy, cholesterol, and potassium contributions were greatest for the non-Hispanic white and black compared with Mexican American children while calcium and vitamin D contributions from the “ready-to-eat processed foods” were greater for non-Hispanic white compared with non-Hispanic black and Mexican American children. Fiber intake from the category was greater for non-Hispanic white compared with non-Hispanic black and added sugar intake was greater for non-Hispanic black compared with Mexican American children. Nutrient intake differences from foods from restaurants/cafeterias among race/ethnic groups were not observed.

## 4. Discussion 

All categories of foods contributed a variety of nutrients to the diets of US children and contributions by category were neither of consistently high nutritional quality (*i.e*., high in dietary components to increase and low in dietary components to decrease as per the 2010 DGA) nor uniformly of low nutritional quality (*i.e*., low in dietary components to increase and high in dietary components to decrease, Table 1, Table 2 and Table 3 and Supplemental Figure S1). “Minimally processed foods” provided proportionally lower contributions to daily energy intake, added sugars, and sodium intake and higher contributions to the daily intake of several nutrients essential for nutrient adequacy, disease prevention, and overall good health [2] (dietary fiber, vitamin D, calcium, potassium) compared with the other processed food categories and for all race/ethnic groups. However, “minimally processed foods” also contributed high amounts of cholesterol, a nutrient to decrease, compared with the energy contributions of the category. Inclusion of fresh fruits, vegetables, eggs, meats, milk and other nutrient dense foods may explain the unique nutrient profile of this category. These results may provide additional context to a recent longitudinal Brazilian cohort study (*n* = 345) among children 3–4 years old and 7–8 years old that documented an evaluation of the contribution of processed and “ultra-processed” food products to child lipid profiles [18]. The authors concluded that “ultra-processed” product consumption (using different categorization criteria) was a predictor of increases in total cholesterol and LDL cholesterol (from a blood draw) from preschool age to school-age [18]. However, the authors did not include all categories of processed foods in the analyses but only the energy from “processed” and “ultra-processed” foods categories. Energy from “minimally processed foods” was not included and may represent a potentially stronger association with blood lipid concentrations. 

Intake from the “mixtures of combined ingredients” category was similarly inconsistent with regard to nutritional quality regarding the proportionally high contributions to sodium and fiber, nutrients to decrease and increase, respectively, and the proportionally low contributions to total and added sugars, nutrients to decrease, and vitamin D and potassium, nutrients to increase. Foods in this category included breads and tortillas; cheeses; condiments, dressings, and sauces; and ready to serve soups, offering a variety of nutrients with diverse contributions to the reported dietary intake of US children. 

The “ready-to-eat processed foods” category, compared with other IFIC Foundation categories, provided proportionally higher contributions to total and added sugars, both nutrients to decrease as per the 2010 DGA, and lower contributions to calcium, vitamin D, and potassium, nutrients to increase. Cholesterol intake, a nutrient to decrease, attributed to this category was low when compared with the contributions of other IFIC Foundation categories. Thus, similar to the other IFIC Foundation categories, the “ready-to-eat processed foods” category demonstrates a “mixed bag” with regard to the nutrients to increase and decrease. “Ready-to-eat processed foods” included sweetened beverages, soft drinks, cookies, cakes, candy, juice drinks, and other foods containing added sugars which may explain the high reported intake of total and added sugars derived from this category. Foods included in this category, such as grain desserts and soda, ranked as top energy contributors among US children age 2–18 years old [19] in previous research and may make little contribution to the nutrients to increase. However, other foods included in this category, such as ready-to-eat cereal, yogurt, and nut butters may make higher contributions to nutrients to increase and other essential nutrients, demonstrating the diversity of nutrient profiles among foods with similar processing levels. 

Differences in covariate-adjusted mean intake of cholesterol, added sugars, calcium, vitamin D, potassium, and fiber were present among race/ethnic groups for all foods and IFIC Foundation category foods but not for food from restaurants/cafeterias (Table 4 and Supplemental Figure S2). In these cases, contributions of the individual IFIC Foundation categories represent a source of overall nutrient difference among the groups. Mexican American children had greater overall intake of cholesterol and fiber compared with non-Hispanic white and black children and lower overall intake of added sugars. Calcium, vitamin D and potassium intake were also higher compared with intakes of non-Hispanic black children. As such, the overall nutrient intake profile of Mexican American children featured higher intakes of many of the nutrients to increase and one nutrient to decrease compared with the other groups. Previous studies found that Mexican American children reported higher total fruit intake compared with non-Hispanic white children [20] and Mexican American and non-Hispanic white children drank significantly more milk than African American children [21]. Milk and minimally processed fruit are included in the “minimally processed foods” category and may help explain the greater energy, fiber, and potassium contributed by this category to Mexican American children’s total dietary intake compared with the other groups. Calcium and vitamin D contributions from this category were also higher among Mexican American compared with non-Hispanic black children. High intake of “minimally processed foods” such as eggs and fresh meat may contribute to high cholesterol intakes among Mexican American compared with non-Hispanic black or white children. 

The pattern of higher cholesterol, calcium, vitamin D, potassium, and fiber intakes among Mexican American compared with non-Hispanic white and black children was repeated in the results of the “mixtures of combined ingredients” category. However, lower energy and intakes of the aforementioned nutrients among Mexican American children compared to at least one of the other race/ethnic groups were observed for the “ready-to-eat processed foods” and “prepared foods/meals”. In addition, Mexican American children were consistently in the group with lower added sugar intake for every IFIC Foundation processed food category where differences were observed among race/ethnic groups. The intake of foods that are rich in the nutrients to increase and contain few of the nutrients to decrease were positive behaviors among Mexican American children but limiting cholesterol laden foods may also improve dietary intake in this group.

Non-Hispanic white children had higher mean intakes of the nutrients to increase, calcium, vitamin D, potassium and fiber compared to non-Hispanic black children, and lower intakes of cholesterol compared with Mexican American children for all foods, IFIC Foundation category foods and “minimally processed foods”. However, non-Hispanic white children also had comparatively higher mean intakes of added sugars compared with Mexican American children from these categories. Foods in the “ready-to-eat processed foods” and “prepared foods/meals” categories, added greater energy and added sugars to non-Hispanic white children’s diets compared with Mexican American children’s diets. The “ready-to-eat processed foods” also contributed more nutrients to increase, calcium and vitamin D to the diets of non-Hispanic white compared with both race/ethnic groups of children, more fiber compared with non-Hispanic black children and more potassium compared with Mexican American children. “Prepared foods/meals” contributed more nutrients to increase, calcium, vitamin D, potassium, and fiber to the diets of non-Hispanic white compared with the Mexican American children. Dietary intake may be improved in non-Hispanic white children by limiting added sugars while maintaining high amounts of nutrients to increase.

Non-Hispanic black children had lower calcium, vitamin D, potassium and fiber compared with non-Hispanic white and Mexican American children from all foods, IFIC Foundation category foods, and “minimally processed foods”. These low intakes of nutrients to increase were paired with higher added sugar intake from all foods and IFIC Foundation category foods when compared with Mexican American children. Sources of added sugar, including sweetened soft drinks and sweets (chocolates and candy), were more frequently consumed among African American 6th–10th grade children compared with white and Hispanic children in a previous study using nationally representative data from the Health Behavior in School-aged Children’s survey (2001–2002, 2005–2006, and 2009–2010) [22]. Cholesterol contributions from all foods and IFIC Foundation category foods, however, were lower among non-Hispanic black compared with Mexican American children. A lower proportion of the non-Hispanic black group compared with the other race/ethnic groups, consumed foods from the “minimally processed foods” and exhibited lower mean energy intake from this processed food category. A previous study reported that African Americans consumed less fruit and dairy than whites and other race/ethnic groups [23]. The “minimally processed foods” category includes several foods fitting this profile. Fruit and dairy may be included in other processed food categories but non-Hispanic black children were consistently in the group of lower intake for calcium, vitamin D, potassium, and fiber regarding the “mixtures of combined ingredients”, and “ready-to-eat processed foods” (except for potassium) compared with either one or both of the other race/ethnic groups. Non-Hispanic black children may improve dietary intake and ameliorate dietary disparities by consuming foods containing more of the nutrients to increase and less of the nutrients to decrease.

The NHANES 2003–2008 is a large, well-designed and executed national survey [24]. Under-reporting of dietary intake is common and may be particularly problematic among children [25]. The foods most often forgotten or under-reported are butter, desserts, and sweet baked goods [26,27] and may result in under-reported intake of nutrients originating from these foods. Although, theoretically, the mean intake represents the mean usual intake of the average person in the group, these data should be interpreted with caution; the diet of an individual child on any one day, are not identical to the mean daily group intake described. Each child in a population-based group may have diverse daily dietary patterns that vary over time. In addition, many other nutrients outside the scope of this study are essential for child growth and development and intake may be more or less contributed by the processed food groups included here. Poverty-income-ratio was controlled in the analysis but residual confounding by family income or parental education may still exist due to the complex nature of these characteristics. Estimates are from cross-sectional data and temporal associations of intake with individual characteristics cannot be inferred.

## 5. Conclusions 

All children, regardless of race or ethnicity consume processed foods. Approximately 66% to 84% of total daily energy, saturated fat, cholesterol, fiber, total sugar, added sugars, calcium, vitamin D, potassium, and sodium intake are contributed by one of the five categories of processed foods that are not obtained from restaurants or cafeterias. Processed foods contribute many of the nutrients needed for healthy child growth and development, yet also contribute to excess intakes of energy and decreased intake of certain nutrients. Dietary recommendations based on the category of food processing are not appropriate due to inconsistent contributions toward the intakes of nutrients to increase and decrease in children’s diets. Dietary recommendations for children to consume a healthy diet should focus on energy and nutrient content, frequency of consumption, and serving size of foods rather than the processing level.

## Supplementary Materials

The following are available online at http://www.mdpi.com/2072-6643/7/12/5503/s1, Figure S1: International Food Information Council Foundation processed food category contributions (%) to the total daily mean (standard error of the mean) number of foods consumed and the total daily mean (standard error of the mean) energy and selected nutrient intake of non-Hispanic white (*n* = 2954), non-Hispanic black (*n* = 3139) and Mexican American (*n* = 3061) US children 2–18 years participating in NHANES 2003–2008. Figure S2: Covariate (Kcal, gender, age, and poverty-income-ratio)-adjusted mean (standard error of the mean) daily percent contribution of International Food Information Council Foundation processed food categories to the daily total percentage of the population consuming foods and intake of energy and selected nutrients by non-Hispanic white (*n* = 2954), non-Hispanic black (*n* = 3139) and Mexican American (*n* = 3061) US children 2–18 years participating in NHANES 2003–2008.
