# Supplementary material for: Processed Food Contributions to Energy and Nutrient Intake Differ among US Children by Race/Ethnicity
Source: Nutrients. 2015 Dec 2;7(12):10076–88. doi: 10.3390/nu7125503 (PMC4690055; doi:10.3390/nu7125503)
Supplement: Supplementary file 1 [file nutrients-07-05503-s001.docx]

Supplementary Materials: Processed Food Contributions to Energy and Nutrient Intake Differ among US Children by Race/Ethnicity

Heather A. Eicher-Miller ^1,^*^,†^, Victor L. Fulgoni, III ^2,†^ and Debra R. Keast ^3,†^


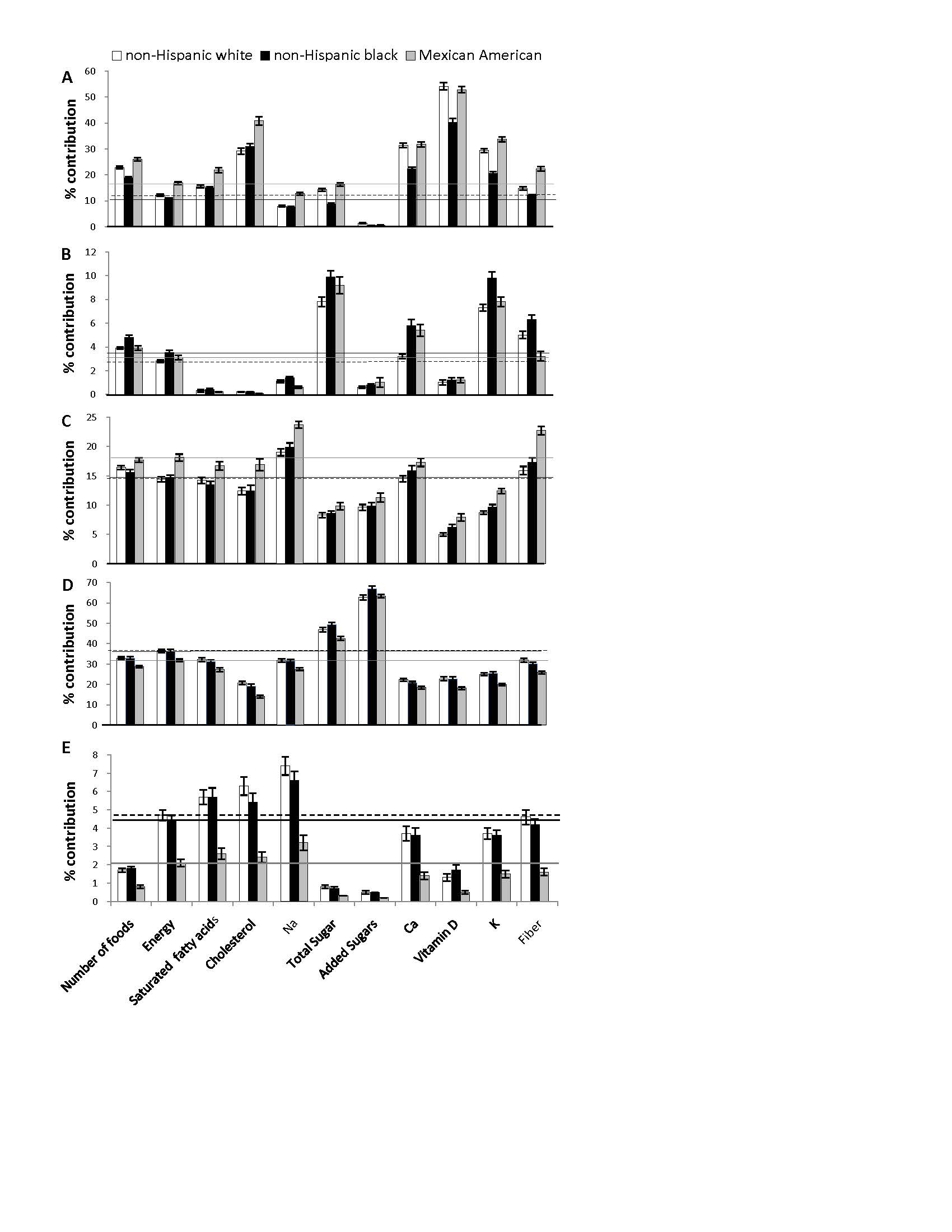


**Figure S1.** International Food Information Council Foundation processed food category contributions (%) to the total daily mean (standard error of the mean) number of foods consumed and the total daily mean (standard error of the mean) energy and selected nutrient intake of non-Hispanic
white (*n* = 2954), non-Hispanic black (*n* = 3139) and Mexican American (*n* = 3061) US children 2–18 years participating in NHANES 2003-2008. Scales showing percent contributions on the y-axis are varied in order to show contributions of each processed food category. (**A**) minimally processed; (**B**) processed for preservation; (**C**) mixtures of combined ingredients; (**D**) ready-to-eat; (**E**) and prepared foods/meals; % energy contributions represented by dashed (non-Hispanic white), black (non-Hispanic black), and grey (Mexican American) lines. Bars=mean (standard error of the mean).


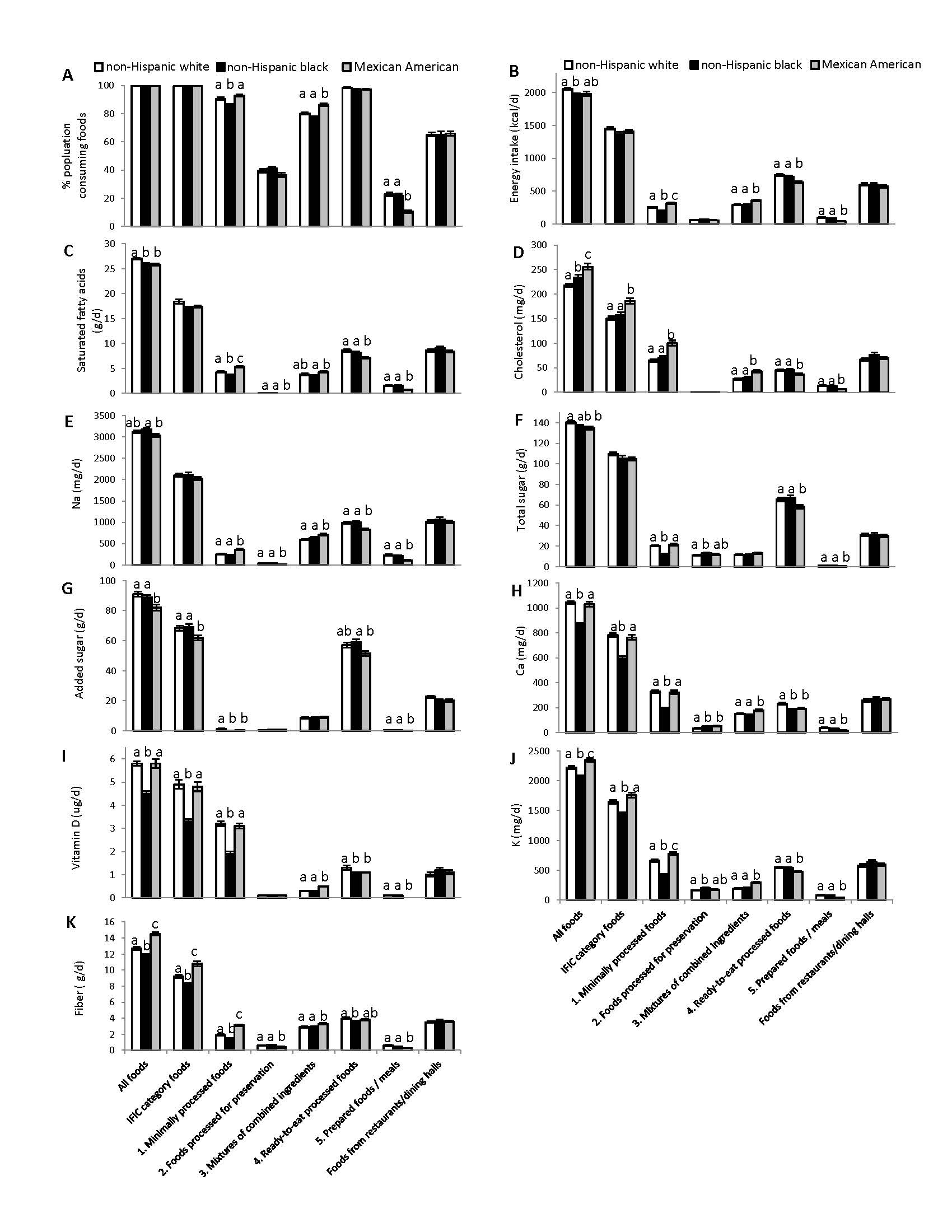


**Figure S2.** Covariate (Kcal, gender, age, poverty-income-ratio)-adjusted mean (standard error of the mean) daily percent contribution of International Food Information Council Foundation processed food categories to the daily total percentage of the population consuming foods and intake of energy and selected nutrients by non-Hispanic white (*n* = 2954), non-Hispanic black (*n* = 3139) and Mexican American (*n* = 3061) US children 2–18 years participating in NHANES 2003–2008. Significant differences (*p* < 0.05/3 race/ethnic groups, Bonferronni type adjustment for multiple comparisons of sub-groups) within each category of foods are indicated by differing lower-case alphabetic superscript (**a,b,c**); significant differences are not established for race/ethnic sub- groups with a similar lower-case alphabetic superscript.
